# Disulfide Cross-Linked Thiolated Chitosan/Oxidized Dextran–Oleylamine Micelle–Hydrogel System with pH/ROS Dual-Responsive Cascade Drug Release for Integrated Colon Targeting and Mucosal Repair in Inflammatory Bowel Disease

**DOI:** 10.3390/pharmaceutics18050602

**Published:** 2026-05-14

**Authors:** Jiangtao He, Chunyu Gan, Tongxia Chi, Jia Liu, Tuya Bai, Xin Wu, Guodong Liang, Ruijuan Li, Yuheng Ma

**Affiliations:** 1 College of Pharmacy, Inner Mongolia Medical University, Hohhot 010110, China; 15525591427@163.com (J.H.); 13889254290@163.com (C.G.); 2023110075@stu.immu.edu.cn (T.C.); 20090010@immu.edu.cn (J.L.); 20120019@immu.edu.cn (T.B.); 20220821@immu.edu.cn (G.L.); 2 Inner Mongolia Autonomous Region Engineering Research Center of New Pharmaceutical Screening, Inner Mongolia Medical University, Hohhot 010110, China; 3 Shanghai Wei Er Lab, No. 358, Tian Chen Road, Qingpu District, Shanghai 201707, China; baolong@shblyy.com

**Keywords:** inflammatory bowel disease, oral colon-targeted delivery, polymeric micelles, mucoadhesive hydrogel, mucosal repair

## Abstract

**Background:** Oral colon-targeted delivery for inflammatory bowel disease (IBD) faces significant challenges, including limited gastrointestinal stability, premature drug release, and insufficient mucosal retention. **Methods:** To address these limitations, a mucoadhesive polysaccharide-based composite hydrogel incorporating prednisolone-loaded polymeric micelles was developed to enhance colonic delivery and promote mucosal repair. Amphiphilic oxidized dextran–oleylamine (ODEX-OA) copolymers were synthesized to self-assemble into prednisolone-loaded micelles. These micelles were subsequently embedded within a thiolated chitosan (CSSH) hydrogel through a Schiff base reaction, yielding the ODEX-OA-Pred-CSSH composite. The resulting system was comprehensively characterized for particle size, mucoadhesion, degradation, and pH/ROS dual-responsive drug release. Its colon-targeting capability and therapeutic efficacy were subsequently assessed in a dextran sulfate sodium (DSS)-induced colitis mouse model. **Results:** In vitro, the composite hydrogel demonstrated nanoscale micellar size, enhanced drug release kinetics under simulated inflammatory colonic conditions, and prolonged colonic retention for up to 24 h following oral administration. In vivo, studies confirmed that ODEX-OA-Pred-CSSH significantly alleviated colitis, evidenced by a reduced disease activity index, diminished pro-inflammatory cytokine levels, restored colon length, decreased spleen index, and improved histological mucosal repair. **Conclusions:** These findings collectively suggest that this mucoadhesive micelle–hydrogel composite represents a promising and effective oral colon-targeted platform for the treatment of IBD.

## 1. Introduction

Inflammatory bowel disease (IBD), primarily encompassing ulcerative colitis (UC) and Crohn’s disease (CD), is a chronic, relapsing inflammatory disorder characterized by abdominal pain, diarrhea, weight loss, intestinal barrier dysfunction, and hematochezia [1]. Its pathogenesis is intricately linked to gut microbial dysbiosis, mucosal barrier disruption, immune dysregulation, and oxidative stress [2,3]. Persistent inflammation induces excessive reactive oxygen species (ROS) production in the intestinal mucosa, thereby aggravating tissue injury and perpetuating the inflammatory cascade. Consequently, effective IBD therapies must not only suppress inflammation but also promote mucosal repair. Current clinical treatments, including glucocorticoids, aminosalicylates, immunosuppressants, antibiotics, and biologics, are often limited by systemic adverse effects, drug resistance, and poor compliance during long-term administration [4,5,6]. Oral colon-targeted drug delivery systems (CDDSs) have therefore garnered increasing attention due to their ability to enhance local drug accumulation at inflamed sites while minimizing systemic exposure [7,8,9]. Leveraging the unique colonic environment, characterized by variations in pH, microbiota, and mucosal status, a range of stimulus-responsive delivery systems has been developed for IBD treatment [10,11]. Among these, multi-responsive platforms hold particular promise for their capacity to improve targeting precision and overcome the inherent limitations of single-trigger systems [12].

Polymeric micelles are widely utilized as nanocarriers due to their ability to enhance the solubility and stability of poorly water-soluble drugs [13,14,15,16]. Dextran (DEX), a polysaccharide rich in hydroxyl groups, possesses excellent biocompatibility. Its facile modifiability and susceptibility to degradation by colonic microbial enzymes make it an ideal material for micelle construction [17,18]. Specifically, the conjugation of DEX with oleylamine (OA) yields amphiphilic polymers capable of self-assembling into drug-loaded micelles.

However, orally administered micelles remain susceptible to premature disassembly in the gastrointestinal tract, potentially leading to premature drug leakage and diminished delivery efficiency. Furthermore, limited mucosal retention at inflamed sites can further compromise therapeutic efficacy [19,20]. Hydrogels offer an effective strategy to circumvent these limitations. Their high-water content, excellent biocompatibility, tunable degradability, and mucoadhesive properties enable the protection of encapsulated nanocarriers during gastrointestinal transit, prolonged retention at lesion sites, and facilitation of mucosal healing [21,22,23,24,25].

Natural polysaccharides are particularly well-suited for hydrogel construction owing to their excellent biocompatibility, mucoadhesion, and enzyme-responsive degradation within the colon [26,27,28,29]. Among these, thiolated chitosan exhibits enhanced mucoadhesion via thiol-mediated interactions with mucins, potentially further prolonging retention at inflamed colonic sites [30,31].

In this study, we developed an oral colon-targeted composite hydrogel by integrating prednisolone-loaded polymeric micelles within a mucoadhesive polysaccharide hydrogel matrix. Oxidized dextran (ODEX) was conjugated with OA to form an amphiphilic polymer, which subsequently self-assembled into micelles for prednisolone loading (ODEX-OA-Pred). These micelles were then embedded into a hydrogel network formed via a Schiff base reaction between ODEX and thiolated chitosan (CSSH), resulting in the composite hydrogel, ODEX-OA-Pred-CSSH. This system was designed to improve gastrointestinal stability, enhance colonic retention, and achieve pH/ROS dual-responsive drug release. Its degradation behavior, colon-targeting capability, and therapeutic efficacy were subsequently evaluated under simulated gastrointestinal conditions and in a dextran sulfate sodium (DSS)-induced colitis mouse model.

## 2. Materials and Methods

### 2.1. Materials

Dextran (DEX, Mw 40,000), Chitosan (CS, Mw ≈ 200 kDa, degree of deacetylation 80.0–90.0%), oleylamine (OA), and sodium cyanoborohydride (NaCNBH_3_) were purchased from Aladdin Technology Co., Ltd. (Shanghai, China). Sodium periodate (NaIO_4_) was purchased from Inno Chem Technology Co., Ltd. (Beijing, China). N-acetyl-L-cysteine (NAC) and 5,5′-dithiobis(2-nitrobenzoic acid) (DTNB) were purchased from Bidepharm Technology Co., Ltd. (Shanghai, China). Prednisolone (Pred) was obtained from Ouhe Technology Co., Ltd. (Beijing, China). 1,1′-dioctadecyl-3,3,3′,3′-tetramethylindotricarbocyanine iodide (DiR) was obtained from Beyotime Biotech Inc. (Shanghai, China). Dextran sulfate sodium (DSS, Mw 36,000–50,000) was purchased from Yeasen Biotechnology Co., Ltd. (Shanghai, China). Sulfasalazine (SASP) was purchased from Xinyi Tianping Pharmaceutical Co., Ltd. (Shanghai, China). A fecal occult blood test kit (300T) was purchased from Yuanye Biotechnology Co., Ltd. (Shanghai, China). ELISA kits for TNF-α, IL-1βand IL-10 were obtained from Solarbio Biological Reagents Co., Ltd. (Beijing, China). The 4% paraformaldehyde fixative was purchased from Senbeijia Biotechnology Co., Ltd. (Nanjing, China). Unless otherwise specified, all other reagents were of analytical grade and used as received, and ultrapure water was used throughout all experiments.

### 2.2. Synthesis and Characterization of ODEX

Three 2.5 g portions of dextran (DEX) were separately dissolved in 50 mL of ultrapure water in 100 mL round-bottom flasks at 50 °C with magnetic stirring. After complete dissolution and cooling to room temperature, sodium periodate (NaIO_4_; 1.6, 3.2, or 4.8 g) was added to each flask to yield oxidized dextran samples with varying degrees of oxidation. Reactions were conducted in the dark for 4 h, then quenched by the dropwise addition of 1 mL of glycerol, followed by 15 min of additional stirring.

The reaction mixture was transferred to a dialysis bag (MWCO 3.4 kDa) and dialyzed against deionized water for 3 days, with the external water replaced 3–4 times daily to remove residual reagents and by-products. Impurity removal was verified by testing the dialysate with a 1% silver nitrate (AgNO_3_) solution; the absence of precipitate confirmed thorough purification. After dialysis, the purified solutions were frozen at −80 °C and lyophilized to yield white, fibrous oxidized dextran (ODEX) powders. The product was stored at −20 °C in a dark place until required.

The obtained samples were denoted as ODEX-L, ODEX-M, and ODEX-H, corresponding to their low, medium, and high degrees of oxidation, respectively.

The chemical structures of DEX, ODEX-L, ODEX-M, and ODEX-H were characterized by Fourier transform infrared spectroscopy (FT-IR) in the range of 4000–500 cm^−1^. Proton nuclear magnetic resonance (^1^H NMR) spectra of DEX and ODEX were recorded on a 600 MHz NMR spectrometer. Briefly, 4 mg of each sample was dissolved in a deuterated solvent mixture 200 µL of deuterated chloroform (CDCl_3_) and 550 µL of deuterated dimethyl sulfoxide (DMSO-d_6_)), and the spectra were then collected.

The degree of oxidation of ODEX was determined by titration with hydroxylamine hydrochloride. To prepare the hydroxylamine hydrochloride reagent solution: Dissolve 580 mg of hydroxylamine hydrochloride in ultrapure water. Then, add 1 mL of a 0.05% (*w*/*v*) methyl orange solution dilute the mixture to 100 mL, and adjust the pH to 4.0 using sodium hydroxide.

For the titration, 100 mg of ODEX (100 mg) was dissolved in 25 mL of the prepared reagent solution and stirred at room temperature for 5 h. As a control, 100 mg of unmodified DEX was treated under the same conditions. The resulting solution was then titrated with 0.1 mol/L standard sodium hydroxide solution until its color changed from red to yellow and remained stable for 30 s.

The difference in NaOH consumption between the ODEX sample and the DEX control was recorded as ΔV. The oxidation degree (η) was calculated according to Equation (1):

*η* = 162 × ∆*V* × *C* × 10 − 32*W* (1)

where ΔV is the volume of NaOH solution consumed (mL), C is the concentration of NaOH solution (mol/L), W is the mass of ODEX (g), and 162 is the molecular weight of the dextran repeating unit (g/mol).

### 2.3. Synthesis and Characterization of ODEX-OA

ODEX (100 mg) was dissolved in 40 mL of ultrapure water. Separately, oleylamine (OA, 0.16 g) was dissolved in 2 mL of ethanol with vigorous stirring. The OA solution was then added dropwise to the ODEX solution, and the mixture was sonicated for 5 min to ensure uniform dispersion. Sodium cyanoborohydride (NaCNBH_3_, 0.096 g) was then added, and the reaction mixture was shaken at 100 rpm at 37 °C for 24 h. During this reaction, the aldehyde groups of ODEX reacted with the amino groups of OA via reductive amination, yielding the hydrophobically modified derivative ODEX-OA.

Once the reaction was complete, the crude product was precipitated by adding ethanol and allowed to stand for 1 h. The mixture was then centrifuged, and the precipitate was collected. The precipitate was repeatedly washed with methanol and ultrapure water to remove unreacted OA and other residual reagents. Finally, the purified product was freeze-dried to yield ODEX-OA.

The chemical structure of ODEX-OA was characterized by FT-IR over the range of 4000–500 cm^−1^.

### 2.4. Preparation and Characterization of ODEX-OA-Pred Micelles

#### 2.4.1. Preparation of ODEX-OA-Pred Micelles

Pred (2 mg) was initially dissolved in 10 mL of HPLC-grade methanol, then ODEX-OA (10 mg) was added. The resulting blend was sonicated for 30 min to achieve a uniform dispersion. The organic solvent was subsequently removed via rotary evaporation at 68 °C under vacuum for 10 min, yielding a thin film on the flask interior. This film was then gently hydrated with 10 mL of pre-warmed ultrapure water at 50 °C for 20 min. The resulting dispersion was centrifuged at 3500 rpm for 10 min to eliminate free drug molecules and oversized particulates. The collected supernatant was then filtered through a 0.45 μm pore-size membrane to produce drug-encapsulated micelles (ODEX-OA-Pred). Blank micelles (ODEX-OA) were fabricated identically, excluding Pred from the formulation.

#### 2.4.2. Determination of the Encapsulation Efficiency and Drug Loading Capacity of ODEX-Pred-OA Micelles

Prepare Pred standard solutions in mobile phase at concentrations of 5.0 µg/mL, 10.0 µg/mL, 20.0 µg/mL, 40.0 µg/mL, 60.0 µg/mL, 80.0 µg/mL and 100.0 µg/mL. Filter through a 0.22 µm microporous filter membrane prior to analysis.

Perform linear regression between peak area (Y) and Pred concentration (X) to plot the standard curve. Pred content was determined by high-performance liquid chromatography (HPLC) according to the method specified in the 2020 edition of the Chinese Pharmacopoeia, under the following conditions: the column was a C18 (250 mm × 4.6 mm, 5 µm, Agilent, Santa Clara, CA, USA); Mobile phase: methanol–water (65:35, *v*/*v*); Detection wavelength: 243 nm; Column temperature: 30 °C; Flow rate: 1.0 mL/min; Injection volume: 10 μL.

The freshly prepared ODEX-Pred-OA solution was initially pre-frozen at −80 °C, followed by lyophilization in a freeze-dryer. The freeze-dryer (Tokyo Rikakiki Co., Ltd., Bunkyo-ku, Tokyo, Japan model FDU-2110) was operated at a vacuum level of 10 Pa, an approximate temperature of −88 °C, and a duration of 24 h; these parameters were applied to all subsequent freeze-drying operations. An accurately weighed portion of the lyophilized product was dispersed in methanol at a 1:4 mass-to-volume ratio, sonicated for 10 min, and the supernatant was subsequently collected and passed through a 0.22 μm microporous membrane. HPLC analysis was performed to determine the peak area, and the obtained values were fitted to the aforementioned standard curve equation to quantify the actual Pred content within ODEX-Pred-OA. Subsequently, encapsulation efficiency (EE) and drug loading capacity (DL) were determined. EE is defined as the proportion of the initial drug amount successfully incorporated into the micelles, whereas DL quantifies the mass percentage of the entrapped drug relative to the total formulation weight.

The mathematical expressions for these parameters are presented below:

Encapsulation efficiency (%) = Actual Pred content in ODEX-Pred-OA/Pred dosage × 100%

Drug loading capacity (%) = Actual Pred content in ODEX-Pred-OA/Total mass of ODEX-Pred-OA × 100%

#### 2.4.3. Characterization of ODEX-Pred-OA Micelles

Micellar morphology was characterized via transmission electron microscopy (TEM). The experimental protocol is summarized as follows: the micellar dispersion was appropriately diluted, and a droplet was deposited onto a copper grid. After ambient-temperature desiccation, TEM imaging was conducted to visualize the sample.

Weigh out 5.0 mg of drug-loaded micelles and dissolve them in 5 mL of ultrapure water. Using a dynamic light scattering (DLS) particle size analyser, determine the hydrodynamic diameter and polydispersity index (PDI) of the micelles. Accurately weigh an additional 5.0 mg of drug-loaded micelles, disperse them in 10 mM PBS (pH 7.4) to a final concentration of 0.1 mg/mL, and subsequently measure their zeta potential.

To assess storage stability, micellar dispersions were stored in triplicate for 7 days at 4 °C and 37 °C. Daily particle size measurements were subsequently conducted using the identical instrument.

### 2.5. Synthesis and Characterization of Thiolated Chitosan (CSSH)

Chitosan (CS, 1.0 g) was dispersed in 92 mL of deionised water in a 250 mL beaker and stirred. HOBt (788 mg) was then added, and stirring continued. N-acetyl-L-cysteine (NAC, 4.5 g) was subsequently introduced, followed by the gradual addition of an aqueous solution of EDC hydrochloride (0.2 M). The pH of the reaction mixture was adjusted to 5.0 using 1 M sodium hydroxide and maintained at this value throughout the reaction. The mixture was then stirred for 3 h at room temperature under light-protected conditions.

The reaction mixture was transferred to a pre-treated dialysis tube (MWCO 12 kDa). It was then dialysed for 36 h, first in 5 mmol/L hydrochloric acid containing 1% sodium chloride, and subsequently for another 36 h in 1 mmol/L hydrochloric acid. The dialysis medium was replaced every 12 h. The purified solution was then lyophilised at −80 °C to obtain thiolated chitosan (CSSH), which was stored at 4 °C in a vacuum under light-protected conditions.

The chemical structures of CS and CSSH were characterized by FT-IR (4000–500 cm^−1^) and proton nuclear magnetic resonance spectroscopy (^1^H NMR, 600 MHz).

#### 2.5.1. Determination of Thiol Content

##### Preparation of Ellman’s Reagent and Phosphate Buffer (0.5 M, pH 8.0)

(1) Preparation of phosphate buffer (0.5 M, pH 8.0)

Solution A was prepared by dissolving 17.91 g of Na_2_HPO_4_·12H_2_O in 100 mL deionised water. Separately, 0.78 g of NaH_2_PO_4_·2H_2_O was dissolved in 10 mL deionised water to generate Solution B. These two solutions were subsequently combined at a volumetric ratio of 94.7:5.3 (A:B), yielding a phosphate buffer with a concentration of 0.5 M and a pH of 8.0.

(2) Preparation of 0.03% Ellman’s reagent solution

DTNB [5,5′-dithiobis(2-nitrobenzoic acid), 0.03 g] was dissolved in the above-prepared phosphate buffer and diluted to 100 mL. The resulting solution was mixed thoroughly and stored in the dark until use.

##### Preparation of the NAC Standard Curve by Ellman’s Assay

N-acetyl-L-cysteine (NAC, 0.408 g) was accurately weighed and dissolved in deionized water in a 10 mL volumetric flask to prepare solution C. A 0.5 mL aliquot of this stock solution was then transferred to a 50 mL volumetric flask and brought to volume with deionized water to produce solution D. Finally, 1 mL of solution D was further diluted with deionised water to a total volume of 10 mL, generating the NAC standard solution.

Aliquots of the diluted NAC standard solution were dispensed into eight individual 10 mL centrifuge tubes at varying volumes (0.2, 0.4, 0.6, 0.8, 1.0, 1.2, 1.4, and 1.6 mL). Correspondingly, phosphate buffer (0.5 M, pH 8.0) was added in volumes of 2.8, 2.6, 2.4, 2.2, 2.0, 1.8, 1.6, and 1.4 mL to bring the total volume in each tube to 3.0 mL. Freshly prepared Ellman’s reagent (0.03% (*w*/*v*) DTNB in phosphate-buffered saline, 3.0 mL) was then introduced into each tube. After thorough vortex agitation, the resultant mixtures were incubated in darkness for 2 h.

Following the incubation period, the reaction mixtures were centrifuged at 4000 rpm for 10 min at 25 °C. The resulting supernatants were harvested, and their absorbance values were determined at 412 nm using a UV-visible spectrophotometer. The calibration curve was established by correlating absorbance readings with corresponding NAC concentrations.

##### Determination of Thiol Content in CSSH

A 20 mg aliquot of lyophilized CSSH was precisely weighed into a 10 mL centrifuge tube and dispersed in 8 mL of deionized water. The sample was allowed to hydrate for 30 min under gentle agitation. Prior to sampling, the dispersion was vortexed to ensure homogeneity. 1 mL of the hydrated CSSH dispersion was then transferred to a new tube and mixed with 2 mL of phosphate buffer (0.5 M, pH 8.0). Subsequently, 3 mL of freshly prepared Ellman’s reagent solution [0.03% (*w*/*v*) DTNB in phosphate buffer] was added. Following thorough vortexing, the mixture was incubated with shaking at 100 rpm at 25 °C in the dark for 2 h.

Following centrifugation at 4000 rpm for 10 min at 25 °C, the supernatant was harvested and its absorbance was determined at 412 nm via UV-Vis spectrophotometry. The thiol content of CSSH was subsequently determined using a pre-established NAC calibration curve and expressed as μmol per gram of polymer.

### 2.6. Preparation and Structural Verification of ODEX-CSSH

The ODEX-CSSH hydrogel was fabricated via Schiff base cross-linking between aldehyde moieties on ODEX and amino functionalities on CSSH. Briefly, ODEX (20 mg) was dissolved in 0.5 mL of 0.01 M PBS (0.01 M, pH 7.2–7.4) under magnetic stirring. Concurrently, CSSH (20 mg) was solubilized in 0.5 mL of 0.5% (*v*/*v*) aqueous acetic acid solution with continuous agitation. To eliminate entrapped air, the CSSH solution was centrifuged at 2000 rpm for 15 min. The two precursor solutions were subsequently combined in equal volumes (0.5 mL each) and immediately vortex-mixed to initiate the gelation process, resulting in the formation of the ODEX-CSSH hydrogel. The resulting material was then transferred to a small vial and allowed to rest. Gel formation was confirmed by its non-flowing state upon vial inversion.

The chemical structures of ODEX, CSSH, and ODEX-CSSH were characterized by FT-IR over a scanning range of 4000–500 cm^−1^.

### 2.7. Preparation and Structural Verification of ODEX-OA-Pred-CSSH

#### 2.7.1. Determination of Hydrogel Formation and Optimal Component Ratio

The freshly prepared ODEX-OA-Pred micellar solution, prepared using the thin-film hydration method described above, was stored at 4 °C until use. ODEX and CSSH solutions were prepared as described for the blank hydrogel. The two solutions were then mixed in different volume ratios (ODEX:CSSH = 2:1, 1:1, 2:3, 1:2, and 2:5), while maintaining a constant total volume. Before gelation, 0.3 mL of the ODEX-OA-Pred micellar solution was added to the precursor mixture and vortexed to obtain the ODEX-OA-Pred-CSSH hydrogel. The gelation time of each formulation was recorded to determine the optimal ODEX-to-CSSH ratio for subsequent experiments.

Gelation time was determined using the inverted vial method and defined as the time required for the precursor solution to lose fluidity upon vial inversion.

#### 2.7.2. Scanning Electron Microscopy (SEM)

The SEM was used to observe the porous microstructures of the blank hydrogel (ODEX-CSSH) and the micelle-loaded hydrogel (ODEX-OA-Pred-CSSH). Freshly prepared hydrogel samples were allowed to stabilize before being completely freeze-dried using a lyophilizer. The freeze-dried samples were then mounted onto an aluminum substrate with a conductive adhesive and subsequently gold-plated by sputtering for 45 s to enhance surface conductivity. Finally, the samples were examined by SEM for morphological characterization.

#### 2.7.3. In Vitro Release of ODEX-OA-Pred-CSSH

To evaluate the release behavior of Pred from ODEX-OA-Pred-CSSH under different gastrointestinal conditions, four release media were used: pH 1.2 (simulating gastric fluid), pH 6.8 (simulating the small intestinal environment), pH 7.4 (simulating the colonic environment,) and pH 7.4 containing 0.5 mM H_2_O_2_ (simulating the inflamed colonic microenvironment with elevated reactive oxygen species (ROS). The release of Pred from ODEX-OA-Pred-CSSH was initially investigated separately in each medium, followed by a sequential release study simulating gastrointestinal transit.

For the pH/ROS-responsive release investigation, fresh ODEX-OA-Pred-CSSH was enclosed within a dialysis membrane and submerged in 100 mL of the corresponding release medium. Experiments were conducted at 37 °C with constant shaking at 100 rpm. At designated time points, 1 mL aliquots were withdrawn for analysis, and an equivalent volume of fresh medium was immediately replenished. Pred levels in the collected samples were quantified via HPLC for subsequent computation of cumulative drug release profiles.

In simulated gastrointestinal transit studies, ODEX-OA-Pred-CSSH, encapsulated within dialysis bags, was sequentially transferred into different release media under the following conditions:

pH 1.2 for 0–2 h, pH 6.8 for 2–6 h, and pH 7.4 for 6–16 h. All experiments were conducted at 37 °C and 100 rpm. At predetermined time points, aliquots were collected and analysed by HPLC to determine the Pred concentration.

The cumulative release rate (%) of Pred was calculated using the following formula:

$$Q\;(\%)=\frac{Cn+\sum C_{n-1}}{L}\times 100\%$$

where Q represents the cumulative release rate (%); C_n_ and C_n−1_ denote the Pred concentrations (mg/mL) in the release medium at the nth and (n−1)th sampling points, respectively; n is the number of sampling events; and L is the amount of Pred loaded in the hydrogel (mg).

#### 2.7.4. In Vitro Degradation of ODEX-OA-Pred-CSSH

The in vitro degradation behaviour of ODEX-OA-Pred-CSSH was evaluated through two distinct assessments: mass loss and prednisolone degradation. For mass loss evaluation, 50 mg hydrogel samples were immersed in either a pH 7.4 buffer or a pH 7.4 buffer containing 0.5 mM H_2_O_2_, and incubated at 37 ± 0.5 °C. At predetermined time points (0, 3, 6, 9, 12, 24, 36, 48, 72, and 96 h), samples were collected, surface moisture was removed, and their wet mass was recorded. To maintain constant experimental conditions, the degradation medium was periodically replaced with fresh buffer. For prednisolone degradation analysis, separate samples of 50 mg free prednisolone and 50 mg ODEX-OA-Pred-CSSH were prepared. These were incubated for 24 h under the same environmental conditions as the mass loss study. Following incubation, the concentrations of prednisolone in both the ODEX-OA-Pred-CSSH samples and the free prednisolone samples were determined by HPLC, allowing for the separate calculation of their degradation rates. All experiments were repeated three times.

The degradation rate was calculated according to the following equation:

$$Degradation\;rate\;(\%)=\frac{W_{0}-W_{i}}{W_{0}}\times 100\%$$

where W_0_ is the initial mass of the hydrogel and W_i_ is the remaining mass at time point “i”.

### 2.8. Animal Experiments

#### 2.8.1. Animals and DSS-Induced Colitis Model

Male BALB/c mice (20–22 g) from Beijing Vital River were housed at Inner Mongolia Medical University’s Laboratory Animal Center with ad libitum food and water. All animal procedures were approved by the Medical Ethics Committee of Inner Mongolia Medical University. Animals acclimatized for one week before experimentation.

Acute colitis was induced by providing 3.0% (*w*/*v*) dextran sulfate sodium (DSS) in drinking water.

#### 2.8.2. In Vivo Colonic Targeting and Retention of ODEX-OA-Pred-CSSH

Colonic targeting and retention of DiR-labelled ODEX-OA-Pred-CSSH and ODEX-OA-Pred were assessed using an in vivo imaging platform. DSS-induced colitic and healthy mice were randomly divided into two cohorts (n = 3 per cohort). Both groups received a single oral gavage of the two fluorescent formulations at 0.75 mg/kg. At 3, 6, 12, and 24 h post-dosing, mice were anesthetized and imaged using a small-animal in vivo imaging system.

For ex vivo imaging, parallel mouse groups received DiR-labelled ODEX-OA-Pred-CSSH and DiR-labelled ODEX-OA-Pred via oral gavage. Mice were sacrificed 6, 12, and 24 h post-administration, and colon tissue was collected for ex vivo fluorescence imaging. Quantitative analysis was performed using the in vivo and ex vivo imaging data. All fluorescence images were processed using Living Image 4.7 software.

#### 2.8.3. Gastrointestinal Distribution of Residual Drug After Oral Administration of ODEX-OA-Pred-CSSH

Twenty-five BALB/c mice were acclimatized for 7 days with ad libitum food and water. Before the experiment, mice were fasted for 12 h with ad libitum water. After oral gavage of the drug-loaded micelle–hydrogel composite (180 mg/kg), five mice were sacrificed at 0, 3, 6, 9, and 12 h post-gavage. The abdomen was incised along the midline, and the stomach, small intestine, and colon were removed. Gastrointestinal tissues and contents were collected separately. The drug was extracted from the saline solution using ultrasonication. After centrifugation (10,000 rpm, 10 min, 4 °C), the supernatant was collected, and Pred concentration in tissues and luminal contents determined by HPLC.

#### 2.8.4. Therapeutic Evaluation of Different Formulations in DSS-Induced Colitis Mice

Male BALB/c mice (6–8 weeks, 20–22 g) were acclimatized for 7 days, then randomly allocated into six cohorts (n = 6 per cohort): control, disease model, positive control, drug-loaded micelle–hydrogel composite, drug-loaded micelles, and blank micelle–hydrogel.

Animals in the control cohort received standard drinking water ad libitum; colitis was induced in all remaining cohorts by supplementing their drinking water with 3% DSS for 9 successive days. Commencing on day 7, therapeutic interventions were administered once daily according to group designation. The control and disease model cohorts received equivalent volumes of physiological saline. The positive control cohort received sulfasalazine (SASP, 450 mg/kg). The drug-loaded micelle–hydrogel composite, drug-loaded micelle, and blank micelle–hydrogel cohorts received ODEX-OA-Pred-CSSH (180 mg/kg), the drug-loaded micelle cohort received ODEX-OA-Pred (180 mg/kg), and the blank micelle–hydrogel cohort received ODEX-OA-CSSH (180 mg/kg). All treatments were administered once daily via oral gavage for 5 consecutive days. From the initial day of DSS administration, body mass, stool consistency, and faecal blood were monitored daily to calculate the Disease Activity Index (DAI).

Twenty-four hours post-final administration, all animals were humanely euthanised, and their colons and spleens were excised.

The colonic segment extending from the cecum to the anus was carefully isolated, after meticulously dissecting away mesenteric attachments and adherent adipose tissue. Colonic length was measured. A 1 cm distal segment was fixed in 4% paraformaldehyde for hematoxylin and eosin (H&E) staining and histopathological assessment. The residual colonic tissue was mechanically homogenised, and the resultant supernatant was collected after centrifugation at 10,000 rpm for 10 min and then preserved at −80 °C for further analysis. Concentrations of TNF-α, IL-1β, and IL-10 were quantified using commercial ELISA kits. The spleen was rinsed with ice-cooled physiological saline, gently blotted dry, and weighed. The splenic index was computed as the ratio between splenic mass and total body mass.

All animal experimental protocols were performed in accordance with the ethical guidelines approved by the Ethics Committee of Inner Mongolia Medical University.

### 2.9. Statistical Analysis

Quantitative results are presented as mean ± standard deviation (SD), obtained from at least three independent experimental replicates or observations. Statistical analyses were performed using SPSS 25.0 software (IBM Corp., Armonk, NY, USA). Comparisons between two groups were analyzed using Student’s *t*-test, while multiple-group comparisons were assessed by one-way analysis of variance (ANOVA). Statistical significance was set at *p* < 0.05.

## 3. Results

### 3.1. Synthesis and Characterization of ODEX

**Figure 1:**
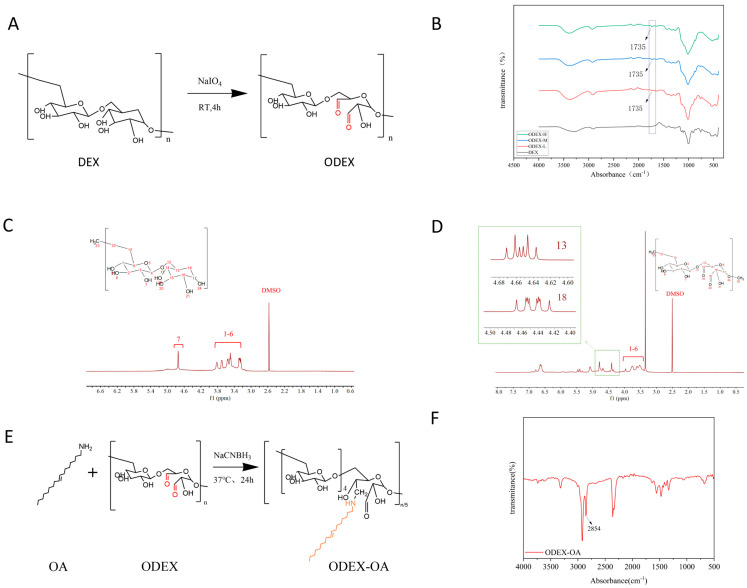
(**A**) Illustration of the synthesis of ODEX. (**B**) FT-IR plots for DEX and ODEX series. (**C**) ^1^H-NMR pattern of DEX. (**D**) ^1^H-NMR Spectra of ODEX. (**E**) Synthesis of ODEX-OA. (**F**) FT-IR plot for ODEX-OA.

Figure 1A illustrates the synthesis of ODEX (oxidized dextran). Three ODEX samples with varying degrees of oxidation were synthesized by adjusting the molar ratio of sodium periodate (NaIO_4_) to dextran monomers. In the FT-IR spectra (Figure 1B), distinct characteristic peaks at 1735 cm^−1^, corresponding to aldehyde/ketone groups, are observed in the three ODEX samples compared to DEX. These peaks intensify with an increasing degree oxidation level, while other original characteristic peaks remain unchanged. This indicates the partial oxidation of hydroxyl groups on DEX to aldehyde groups, reflecting a progressively higher overall oxidation among the three ODEX groups.

In the ^1^H NMR spectra (Figure 1C,D), proton signals in the 3.2–4.0 ppm range are attributed to protons within the dextran ring. Conversely, ODEX exhibits novel hemiacetal signals between 4.2 and 5.6 ppm, thereby corroborating the generation of aldehyde functionalities, in contrast to DEX.

The aldehyde content in ODEX was determined using the hydroxylamine hydrochloride titration method. Three ODEX samples, characterized by varying degrees of oxidation, were synthesized by modulating the molar ratio of the oxidant NaIO_4_ to dextran monomer units. Specifically, when NaIO_4_-to-dextran monomer molar ratios were set at 1.5:1, 3.0:1, and 4.5:1, the achieved oxidation degrees were 34.83 ± 1.10%, 42.12 ± 0.93%, and 60.75 ± 1.50%, respectively. The corresponding products were designated as ODEX-L (low oxidation), ODEX-M (medium oxidation), and ODEX-H (high oxidation). These findings corroborate the successful synthesis of ODEX possessing tunable aldehyde contents, thereby establishing a structural foundation for subsequent conjugation with amino-functionalized molecules.

### 3.2. Synthesis and Characterization of Amphiphilic Polymer ODEX-OA

To render ODEX amphiphilic for hydrophobic drug encapsulation, oleylamine (OA) was grafted onto its aldehyde groups via a Schiff base reaction, as illustrated in Figure 1E. The FT-IR spectrum of ODEX-OA (Figure 1F) exhibited a new absorption peak at 2854 cm^−1^, corresponding to the symmetric stretching vibration of methylene groups (-CH_2_-) in the long alkyl chain of OA, thereby confirming the successful conjugation of OA onto the ODEX backbone. This amphiphilic polymer facilitated the subsequent self-assembly into drug-loaded micelles.

### 3.3. Preparation and Characterization of Drug-Loaded Micelles ODEX-OA-Pred

**Figure 2:**
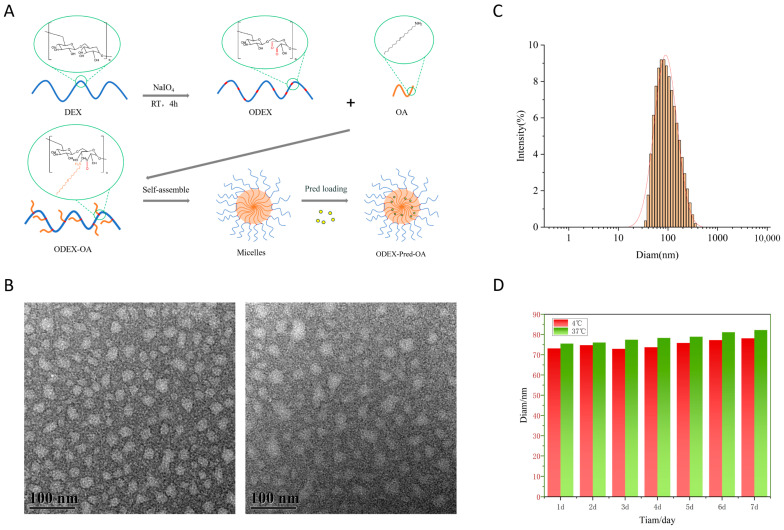
(**A**) The schematic diagram showing the formation of drug-carrying micelles (ODEX-OA-Pred). (**B**) Transmission electron micrographs of ODEX-OA-Pred (**A**) and ODEX-OA (**B**) (scale bar, 100 nm). (**C**) Particle size distribution of ODEX-OA-Pred. (**D**) Stability of ODEX-OA-Pred (n = 3).

The ODEX-OA-Pred drug-loaded polymeric micelles were prepared using the thin-film hydration method. The self-assembly process of the polymeric micelles is illustrated in Figure 2A. ODEX-OA-Pred micelles feature a spherical structure, with a hydrophobic OA segment encapsulating Pred forming the core and the hydrophilic ODEX component constituting the shell. Their drug loading capacity was determined by HPLC to be 12.46%, with an encapsulation efficiency of 81.00%. For the treatment of IBD, the standard oral dose of prednisolone for adults is 20–40 mg/day (during the acute phase). This suggests a required daily intake of approximately 160–320 mg of drug-loaded micelles. When incorporated into a CSSH hydrogel (at a micelle-to-hydrogel matrix ratio of 2:1, *w*/*w*), the total daily dose of the oral formulation becomes approximately 240–480 mg. This dosage is significantly lower than the upper limit for conventional oral solid formulations (<1–2 g), suggesting good patient compliance. TEM images of ODEX-OA-Pred and ODEX-OA (Figure 2B) revealed the micelles to exhibit a near-spherical morphology with a uniform particle size distribution and good structural integrity. Particle size measurements (Figure 2C) revealed an average hydrodynamic diameter of 78.73 ± 2.67 nm. Furthermore, the zeta potential was measured to be −19.88 ± 1.45 mV. As shown in Figure 2D, the ODEX-OA-Pred solution maintained stable particle sizes when stored at both 4 °C and 37 °C for 7 days, indicating excellent colloidal stability.

### 3.4. Synthesis and Characterization of CSSH

**Figure 3:**
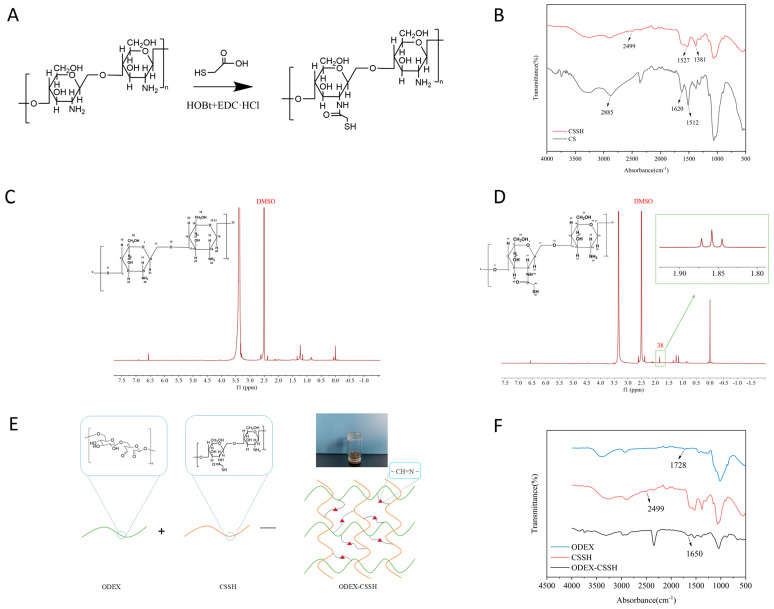
(**A**) Illustration of the synthesis of CSSH. (**B**) FT-IR plots for CS and CSSH. (**C**) 1H-NMR spectrum of CS. (**D**) 1H-NMR pattern of CSSH. (**E**) Schematic diagram of ODEX-CSSH formation. (**F**) FT-IR plot for ODEX-CSSH.

The chemical synthesis reaction formula for thiol chitosan is depicted in Figure 3A. This reaction employs HOBt and EDC·HCl as catalysts. Upon mixing HOBt with the CS solution, it forms a soluble complex with the primary amino groups in the CS molecule, rendering the solution clear and transparent. This interaction simultaneously activates the amino groups, allowing them to rapidly undergo an amidation reaction with the carboxyl groups of NAC to yield thiol chitosan, namely, CSSH.

As shown in Figure 3C,D, the characteristic signals of the sugar rings in CS shifted between 3.0 and 4.0 ppm. The absorption peak at 2.6 ppm corresponds to methylene. Compared to CS, this peak is enhanced in CSSH. A new peak at 1.8 ppm corresponds to the methyl group in CSSH, confirming its successful synthesis. As shown in Figure 3B, the infrared absorption spectrum of CS displays a broad peak near 3200–3500 cm^−1^, corresponding to the stretching vibration of O-H and N-H groups. Peaks between 2800 and 2900 cm^−1^ are attributed to the stretching vibrations of methyl and methylene groups on CS. In the CS molecule, the absorption peak at 1620 cm^−1^ is for the amide I band (C=O), and the peak at 1512 cm^−1^ is for the amide II band (N-H). In the infrared absorption spectrum of CSSH, a new characteristic absorption peak appears near 1527 cm^−1^ for the amide bond, indicating that the carboxyl group of NAC has reacted with the amino group of CS. Additionally, a new absorption peak appears at 2499 cm^−1^, which is the stretching vibration peak of the thiol group. This indicates that the thiol group of NAC has been successfully attached to CS via an amide bond.

Ellman’s reagent method was used to determine the thiol content in the synthesized product. In this experiment, the feeding ratio of CS, NAC, EDC·HCl, and HOBt was 1:4:8:1. The thiol content in CSSH was calculated to be 125.10 ± 2.75 mol/g, providing a quantitative basis for subsequent crosslinking reactions.

### 3.5. Preparation and Structural Verification of Blank Hydrogel ODEX-CSSH

The preparation of the ODEX-CSSH hydrogel is illustrated in Figure 3E. Aldehyde groups on ODEX reacted with amino groups on CSSH through a Schiff base reaction, forming C=N bonds. Simultaneously, thiol groups underwent air oxidation to form disulfide bonds. This dual crosslinking resulted in a robust three-dimensional network structure.

The FT-IR spectrum of ODEX-CSSH is shown in Figure 3F. In the spectrum, the characteristic peak of ODEX at 1728 cm^−1^ vanished following the reaction with CSSH, indicating its consumption during the Schiff base reaction. Conversely, a distinct absorption peak emerged at 1635 cm^−1^ in the ODEX-CSSH spectrum. This peak is characteristic of the amide bond within the Schiff base structure, thereby confirming the successful Schiff base reaction between ODEX and CSSH and the formation of C=N bonds. Furthermore, the weak characteristic peak corresponding to the thiol group at 2499 cm^−1^ in the CSSH spectrum was significantly attenuated or nearly absent in the hydrogel’s spectrum, suggesting its oxidation to form disulfide bonds. Collectively, these spectroscopic findings confirm the successful construction of the ODEX-CSSH hydrogel.

### 3.6. Optimization of Gel-Forming Ratio for ODEX-OA-Pred-CSSH

**Figure 4:**
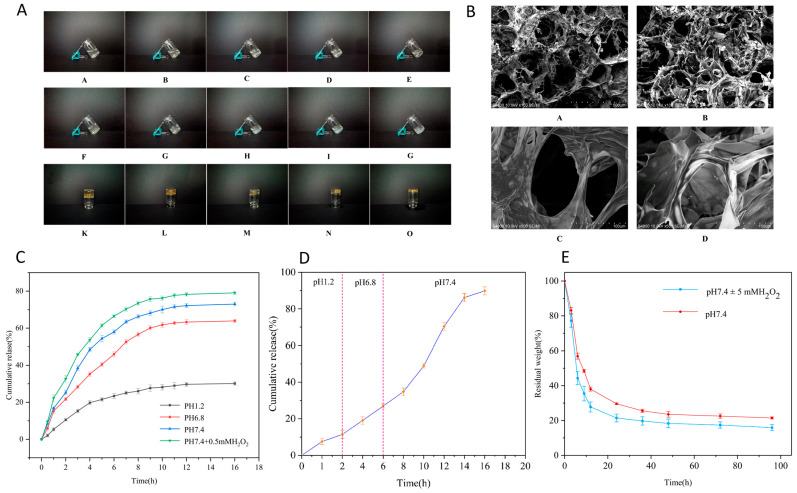
(**A**) Comparison of the controls before and after hydrogel formation (A/B/C/D/E for 2:1, 1:1, 2:3, 1:2, and 2:5 (*v*/*v*) initial mixing; F/G/H/I/J for 2:1, 1:1, 2:3, 1:2, and 2:5 (*v*/*v*) after gel formation; and K/L/M/N/O for 2:1, 1:1, 2:3, 1:2, and 2:5 (*v*/*v*) after 24 h inversion). (**B**) SEM images of hydrogels: (A) ODEX-OA-CSSH (scale bar 500 μm), (B) ODEX-OA-Pred-CSSH (scale bar 500 μm), (C) ODEX-OA-CSSH (scale bar 100 μm), and (D) ODEX-OA-Pred-CSSH (scale bar 500 μm). (**C**) Release profiles of ODEX-OA-Pred-CSSH in different release media (n = 3), pH 1.2 (SGF), pH 6.8 (SIF), pH 7.4 (SCF) and pH 7.4 + 0.5 mM H_2_O_2_ (inflammatory SCF), for 16 h each. (**D**) Release behavior of ODEX-OA-Pred-CSSH in a simulated whole gastrointestinal tract environment (n = 3) at pH 1.2 (SGF) for 0~2 h, at pH 6.8 (SIF) for 2~6 h, and at pH 7.4 (SCF) for 6~16 h. (**E**) Degradation curves of ODEX-OA-Pred-CSSH in media with different pH values (n = 3).

ODEX and CSSH were each prepared at a concentration of 40 mg/mL. When mixed at various ODEX:CSSH volume ratios 2:1, 1:1, 2:3, 1:2, and 2:5 (*v*/*v*), gels formed at room temperature with corresponding gelation times of 30 s, 60 s, 30 min, 12 h, and 12 h, respectively. The hydrogel’s pre- and post-gelation states are shown in Figure 4A. The 2:1 (*v*/*v*) ODEX:CSSH ratio resulted in the shortest gelation time and highest structural integrity. After 24 h, hydrogels formed at this ratio remained stable, exhibiting no flow upon vial inversion. A clear inverse relationship was observed between the ODEX:CSSH volume ratio and gelation time, with decreasing ratios leading to longer gelation times. In contrast, after 24 h, hydrogels prepared with 2:3, 1:2, and 2:5 (*v*/*v*) ratios exhibited slight flow along the vial walls upon inversion. Therefore, the 2:1 (*v*/*v*) ODEX:CSSH ratio was chosen for subsequent experiments to ensure rapid gelation and robust structural stability.

### 3.7. Scanning Electron Microscopy (SEM) Observation

SEM micrographs of ODEX-CSSH and ODEX-OA-Pred-CSSH (Figure 4B) revealed that both hydrogels exhibited numerous pores, indicative of a three-dimensional network structure formed by the intercrossing of ODEX and CSSH molecules. The pore architecture was complex, characterized by the intricate interweaving of relatively large pores with numerous smaller ones. Specifically, in ODEX-OA-Pred-CSSH, drug-loaded micelles were observed embedded within the pores of these network structures. These results collectively indicate the successful formation of interpenetrating three-dimensional network hydrogels from ODEX and CSSH, demonstrating their capacity for drug encapsulation.

### 3.8. In Vitro Release of ODEX-OA-Pred-CSSH

Figure 4C illustrates the release profiles of ODEX-OA-Pred-CSSH across various media. Cumulative drug release was observed to be influenced by both environmental pH and the presence of reactive oxygen species (ROS). After 16 h, the cumulative release rates reached 30.15% at pH 1.2 (simulating gastric fluid), 63.92% at pH 6.8 (simulating small intestinal fluid), 73.03% at pH 7.4 (simulating colonic fluid), and 79.08% at pH 7.4 supplemented with 0.5 mM H_2_O_2_ (simulating inflammatory colonic fluid).

The release behavior of ODEX-OA-Pred-CSSH during simulated gastrointestinal transit is shown in Figure 4D. During the initial 2 h at pH 1.2 (gastric simulation), 18% of Pred was released. Subsequently, an additional approximately 30% was released between 2 and 6 h at pH 6.8 (small intestinal simulation). Upon transition to the colonic environment (pH 7.4), sustained release continued for 10 h, culminating in a final cumulative release exceeding 80%.

These results indicate that the drug-loaded micelle composite hydrogel delivery system exhibits resistance to degradation in acidic environments, enables targeted Pred release in the colon, extends the duration of drug release, and mitigates initial burst release.

### 3.9. In Vitro Degradation of ODEX-OA-Pred-CSSH

As shown in Figure 4E, ODEX-OA-Pred-CSSH exhibited approximately 80% mass loss after 96 h of degradation in a H_2_O_2_-containing buffer medium, with a marginally slower degradation rate observed in a buffer medium without H_2_O_2_. These findings indicate that the hydrogel degrades rapidly in both simulated normal intestinal and colonic environments with elevated ROS levels, demonstrating ROS-responsive degradation. This ROS-responsive degradation behavior consistent, with the drug release profile (Section 3.8), further confirms its potential as an inflammation-responsive delivery system. Furthermore, in in vitro degradation experiments, the degradation rate of free prednisolone in a H_2_O_2_-containing buffer medium exceeded 20%, while that of prednisolone within the ODEX-OA-Pred-CSSH complex was less than 10%. This clearly demonstrates the significant protective effect of the micelles on the drug.

### 3.10. In Vitro Adhesion and In Vivo Retention and Distribution

**Figure 5:**
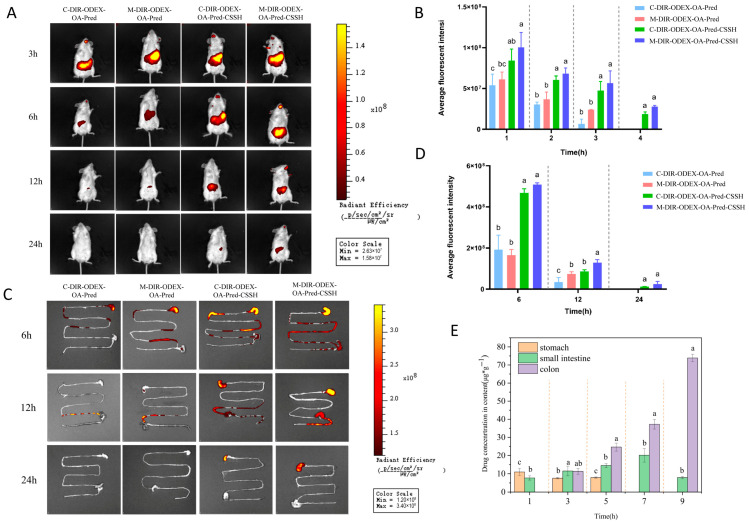
In vivo fluorescence image (**A**) and histogram (**B**) (n = 3). Fluorescence images (**C**) and histograms (**D**) of isolated gastrointestinal tracts (n = 3). (**E**) Analysis of the Pred distribution in various intestinal tissues (n = 5). Note: Different letters represent significant differences (*p* < 0.05).

Figure 5A shows in vivo imaging results. Under the same dosing conditions, DIR-ODEX-OA-Pred micelles exhibited short intestinal retention and rapid fluorescence attenuation in mice, with only weak signals detected at 12 h and complete clearance by 24 h. In contrast, the micelle–hydrogel composite demonstrated stronger retention, with fluorescence still detectable 24 h post-administration. Quantitative analysis in Figure 5B revealed significantly greater fluorescence intensity in the hydrogel composite group than in the micelle group (*p* < 0.05). Furthermore, the hydrogel composite showed stronger fluorescence signals in the inflamed colons of IBD model mice compared to healthy controls at all time points. These results indicate that the hydrogel enhances the targeted adhesion of drug-loaded micelles to the colon; notably, ODEX-OA-Pred-CSSH demonstrated superior adhesion efficacy in inflamed colonic tissues.

Ex vivo colon imaging (Figure 5C,D) further confirmed that drug retention in the hydrogel group was significantly higher than in the micelle group, and retention in the model group far surpassed that in the healthy group, suggesting that the hydrogel improves colonic drug retention through its structural encapsulation and targeting to the inflammatory environment.

Figure 5E shows Pred distribution and content in homogenates from the stomach, small intestine, and colon at different time points. Results showed only trace amounts of Pred in the stomach and small intestine 1 h after intragastric administration. Over time, Pred gradually accumulated in the colon. At 5 h, the PA concentration in the colon slightly exceeded that in both the stomach and small intestine. By 7 h, PA distribution further increased in the small intestine and colon, with no PA detected in the stomach. At 9 h, the PA concentration in the colon was significantly higher than in the stomach and small intestine. These in vivo distribution results indicate that ODEX-OA-PA-CSSH exhibits excellent colon-targeting properties.

### 3.11. In Vivo Therapeutic Effect on DSS-Induced IBD in Mice

The therapeutic effect of ODEX-OA-Pred-CSSH was evaluated in mice with DSS-induced IBD. The impact of ODEX-OA-Pred-CSSH on characteristic IBD symptoms—weight loss, diarrhea, and bloody stools—was assessed by monitoring changes in body weight, stool consistency, and rectal bleeding.

**Figure 6:**
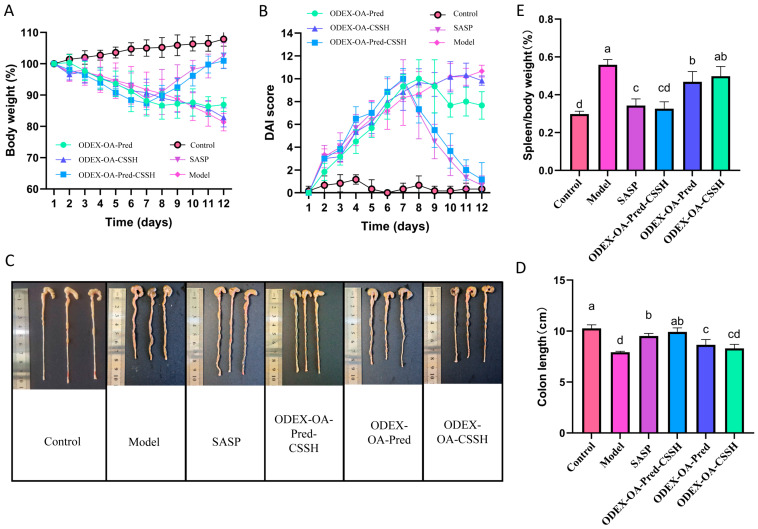
Body weight (**A**). DAI (**B**). A representative image of a colon (**C**). Colon length (**D**). Spleen index (**E**). (n = 6) Note: Different letters represent significance (*p* < 0.05).

During the IBD induction phase (first 9 days), all groups receiving DSS-containing drinking water exhibited rapid weight loss, confirming successful model establishment by day 7 (Figure 6A). After DSS withdrawal on day 9, the rate of weight loss slowed across all groups. However, with the initiation of therapeutic intervention, specifically from day 7, mice in both the SASP and ODEX-Pred-OA-CSSH groups began to regain weight, ultimately restoring normal body weight by the late treatment phase. These results are consistent with expectations, as the composite hydrogel can accumulate in the colon and prolong drug action, both of which contribute to the alleviation of inflammation. Furthermore, fecal analysis revealed that mice in the model group produced unformed stools with grossly visible blood, whereas mice in the ODEX-OA-Pred-CSSH group maintained nearly normal stool consistency and had negative fecal occult blood test results. This correlated with significantly reduced DAIs (Figure 6B), further indicating the therapeutic efficacy of ODEX-Pred-OA-CSSH in IBD management.

Colon length serves as an indicator of colitis severity, as inflammatory scarring at affected sites leads to colonic shortening. Following treatment, mice were euthanized for colon length measurement. As shown in Figure 6C,D, the colon length in the ODEX-OA-Pred-CSSH group (9.9 ± 0.4 cm) did not significantly differ from that of the control group (10.3 ± 0.3 cm) or the SASP group (9.5 ± 0.3 cm). However, it was significantly longer than that observed in the model group (7.9 ± 0.1 cm), ODEX-OA-Pred group (8.7 ± 0.5 cm), and ODEX-OA-CSSH group (8.3 ± 0.4 cm) (*p* < 0.05). The spleen, a primary immune organ rich in lymphocytes and macrophages, plays a crucial role in both cellular and humoral immunity. During inflammatory responses, immune cell accumulation can lead to splenomegaly. As shown in Figure 6E, the spleen index in the ODEX-OA-Pred-CSSH group was significantly lower than that in the model group and closely approximated the control group’s value, though this difference was not statistically significant. These findings indicate that oral administration of ODEX-OA-Pred-CSSH ameliorated colon shortening and mitigated colonic inflammation.

**Figure 7:**
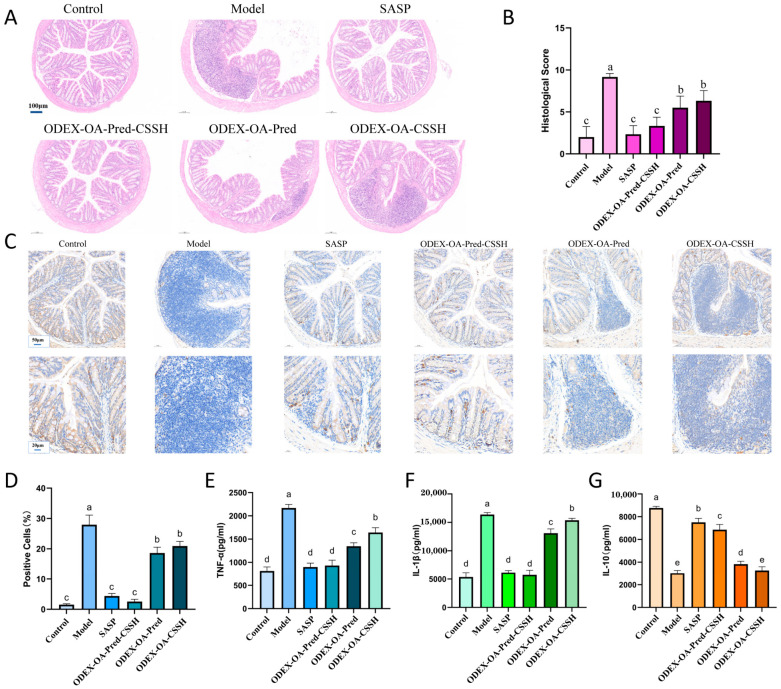
Representative HE-staining images of colon sections (**A**) and histological scores (**B**) (n = 6; scale bar, 100 μm; arrowheads indicate inflammatory cell infiltration, and rectangular boxes indicate structural disorders of the crypts). Analysis plots of MPO immunohistochemical labeling (**C**) and the proportion of MPO-positive cells (**D**) (n = 6) in colonic sections from mice in the indicated groups at 200× (scale bar 50 μm) and 500× (scale bar 20 μm) magnification. Levels of inflammatory cytokines in the colon tissue of mice from the treatment groups ((**E**) TNF-α; (**F**) IL-1β; (**G**) IL-10; n = 6) Note: Different letters represent significant differences (*p* < 0.05).

To evaluate intestinal damage, colon tissues from all groups were subjected to hematoxylin–eosin (HE) staining to assess epithelial injury and inflammatory infiltration. As shown in Figure 7A, control group tissues exhibited intact lamina propria glands, tightly arranged and orderly structures, and normal mucosal architecture. Conversely, model group tissues displayed disrupted epithelial cell structures, disorganized crypts, and extensive inflammatory cell infiltration. HE staining of the ODEX-OA-Pred-CSSH group revealed significant goblet cell hyperplasia, relatively intact and orderly basal crypt structures, and minimal inflammatory cell infiltration, suggesting that ODEX-OA-Pred-CSSH effectively protected intestinal integrity and promoted mucosal repair. Furthermore, compared with the model group, tissues from the ODEX-OA-Pred and DEX-OA-CSSH groups showed partial preservation of intestinal structure; however, residual inflammatory cell infiltration and crypt disorganization remained evident. Histological scoring (Figure 7B) further corroborated these findings, demonstrating significantly lower scores in ODEX-OA-Pred-CSSH-treated mice compared to model mice (*p* < 0.05).

Myeloperoxidase (MPO), Myeloperoxidase (MPO), an abundant neutrophils peroxidase, has potent proinflammatory effects and contributes to tissue damage. MPO expression in colonic tissues was monitored by immunohistochemical (IHC) staining. Figure 7C,D shows the model group displayed the highest MPO expression, followed by the ODEX-OA-CSSH and ODEX-OA-Pred groups. The ODEX-OA-Pred-CSSH and SASP treatment groups exhibited significantly fewer MPO-positive cells than the model group (*p* < 0.05), indicating potent anti-inflammatory effects of ODEX-OA-Pred-CSSH on IBD.

Inflammatory cytokines are critical signaling molecules whose expression levels correlate with IBD severity. Secreted by lymphocytes, monocytes, macrophages, and epithelial cells, these cytokines can be proinflammatory (e.g., TNF-α, IL-1β) or anti-inflammatory (e.g., IL-10). Changes in their secretion levels indicate therapeutic efficacy. Figure 7E,F show DSS significantly increased TNF-α and IL-1β expression in colonic tissue compared to control (*p* < 0.05). ODEX-OA-Pred-CSSH treatment significantly decreased these proinflammatory cytokines to levels not notably different from control or SASP groups. The ODEX-OA-Pred and ODEX-OA-CSSH groups also showed reduced proinflammatory cytokine levels compared to the model. Conversely, DSS markedly decreased IL-10 expression in colonic tissues (Figure 7G), but SASP or ODEX-OA-Pred-CSSH treatment significantly restored its levels. These findings suggest ODEX-OA-Pred-CSSH modulates inflammatory cytokine balance by suppressing proinflammatory factor secretion and increasing anti-inflammatory factor concentrations, demonstrating robust anti-inflammatory activity.

Combining results from histopathology, immunohistochemistry, and cytokine assays revealed that ODEX-OA-Pred-CSSH significantly ameliorates colonic inflammation in IBD mice by modulating inflammatory cytokine balance, reducing oxidative damage, and stimulating mucosal repair, making it a potential candidate for IBD therapy.

## 4. Discussion

This study developed a colon-targeting adhesive, drug-loaded micelle composite hydrogel delivery system Its development involved first synthesizing an amphiphilic polymer, ODEX-OA, by grafting oleylamine (OA) onto oxidized dextran (ODEX). Prednisolone (Pred)-loaded micelles (ODEX-OA-Pred) were then prepared using the film hydration method. Subsequently, the micelles underwent physical crosslinking with thiolated chitosan (CSSH) to form a composite hydrogel (ODEX-OA-Pred-CSSH) with a three-dimensional porous network.

This hydrogel system enhanced Pred’s aqueous solubility, exhibited high drug loading, and uniform particle size. Furthermore, the hydrogel system confers good stability to the ODEX-OA-Pred micelles, although the zeta potential of the ODEX-OA-Pred micelles is below the conventional threshold of ±30 mV, the primary stabilisation mechanism of the ODEX-OA-PA micelles lies in the steric effects generated by the hydrated dextran shell, supplemented by a narrow particle size distribution and a robust self-assembled core–shell structure. Crucially, ODEX-OA-Pred-CSSH showed excellent stability in the harsh gastric environment and enabled intelligent, on-demand drug release, triggered by pH and reactive oxygen species (ROS) in the inflamed colonic microenvironment. In vivo biodistribution studies confirmed its precise colonic targeting capability. In a dextran sulfate sodium (DSS)-induced colitis mouse model, ODEX-OA-Pred-CSSH effectively alleviated colonic inflammation and demonstrated significant therapeutic efficacy. For clinical translation, the materials used in this system are all natural or approved excipients with good biocompatibility, posing a low safety risk. The preparation process employs mild self-assembly and Schiff base cross-linking techniques, requires no complex equipment, and offers potential for large-scale production. Furthermore, the oral route of administration ensures high patient compliance and enables prolonged local action in the colon through mucosal adhesion, thereby reducing systemic exposure and dosing frequency. These advantages collectively demonstrate its potential for clinical translation.

Nevertheless, this system has limitations. Although the DSS-induced acute colitis model rapidly recapitulates key inflammatory features, it does not fully replicate the chronic and relapsing nature of human inflammatory bowel disease (IBD).

Secondly, differences between the animal model and the complex human gastrointestinal environment—such as peristaltic forces, enzymatic diversity, and regional transit times—may impact the performance of this delivery system.

Despite significant progress in nanomedicine for IBD treatment, clinical translation remains constrained. Future research is therefore warranted to elucidate the structure–activity relationship between the physicochemical properties of these polysaccharide-based hydrogels and their in vivo pharmacological behavior. Furthermore, addressing challenges associated with scalable manufacturing and batch-to-batch consistency is essential. With advancements in materials science and the integration of AI-assisted design, this platform may offer a promising avenue for developing next-generation oral therapeutics for IBD.

## Data Availability

The original contributions presented in this study are included in the article; further inquiries can be directed to the corresponding author.
